# Molecular evidence for new sympatric cryptic species of *Aedes albopictus* (Diptera: Culicidae) in China: A new threat from *Aedes albopictus* subgroup?

**DOI:** 10.1186/s13071-018-2814-8

**Published:** 2018-04-04

**Authors:** Yuyan Guo, Zhangyao Song, Lei Luo, Qingmin Wang, Guofa Zhou, Dizi Yang, Daibin Zhong, Xueli Zheng

**Affiliations:** 1 Department of Pathogen Biology, School of Public Health, Southern Medical University, Guangzhou, Guangdong China; 2 Department of Disinfection and Pesticide of Center for Disease Control and Prevention of Guangzhou, Guangzhou, Guangdong China; 3 Department of Epidemiology and Health Statistics, School of Public Health, Guizhou Medical University, Guiyang, Guizhou China; 4 Program in Public Health School of Medicine, University of California, Irvine, California USA

**Keywords:** *Aedes albopictus*, Sympatric cryptic species, *Wolbachia* endosymbiont, Mitochondrial DNA, Genetic diversity, Population structure

## Abstract

**Background:**

*Aedes* (*Stegomyia*) *albopictus* (Skuse) is an indigenous species and the predominant vector of dengue fever in China. Understanding of genetic diversity and structure of the mosquito would facilitate dengue prevention and vector control. Sympatric cryptic species have been identified in the *Ae. albopictus* subgroup in Southeast Asia; however, little is known about the presence and distribution of cryptic species in China. This study aimed to examine the genetic diversity, evaluate potential new cryptic sibling species, and assess the prevalence of *Wolbachia* infections in field populations.

**Methods:**

*Aedes* adult female specimens were collected from five provinces in southern and central China during 2015–2016. Morphological identification was performed under dissection microscope. The mitochondrial DNA cytochrome *c* oxidase subunit 1 (*cox*1, DNA barcoding) locus and the ribosomal DNA internal transcribed spacer region 2 (ITS2) marker were used to examine the genetic variation, evaluate cryptic sibling species, and population structure in the field populations. Screening for the presence of *Wolbachia* was performed using multiplex PCR.

**Results:**

A total of 140 individual specimens with morphological characteristics similar to *Ae. albopictus* were sequenced for DNA barcoding. Among these, 129 specimens (92.1%) were confirmed and identified as *Ae. albopictus*. The remaining 11 specimens, from 2 provinces, were identified as 2 distinct sequence groups, which were confirmed by ITS2 marker sequencing, suggesting the existence of potential cryptic species of *Ae. albopictus*. In *Ae. albopictus*, we found significant genetic differentiation and population structure between populations collected from different climate zones. Medium to high frequencies of *Wolbachia* infections were observed in natural *Ae. albopictus* populations, whereas *Wolbachia* was infrequent or absent in cryptic species populations.

**Conclusions:**

Our findings highlight the population differentiation by climate zone and the presence of novel, cryptic *Aedes* species in China. The low prevalence of *Wolbachia* infections in cryptic species populations could reflect either a recent invasion of *Wolbachia* in *Ae. albopictus* or different host immune responses to this symbiont in the cryptic species. The study provides useful information for vector control and host-symbiont coevolution. Further study is needed to investigate the potential for arbovirus infection and disease transmission in the emerged cryptic species.

**Electronic supplementary material:**

The online version of this article (10.1186/s13071-018-2814-8) contains supplementary material, which is available to authorized users.

## Background

Over the past decades, more than 11 sibling species or cryptic species have been identified and characterized in the *Aedes albopictus* subgroup of the Scutellaris group in the subgenus *Stegomyia* of *Aedes* [1–3]. Of these species, *Ae. albopictus*, originating from Asia, is the most widely distributed and has invaded on every continent except Antarctica [4, 5]. *Aedes albopictus* is considered a medically important species and is a major vector of several human arboviruses, including dengue, Zika, chikungunya, yellow fever and West Nile viruses [5–10]. Dengue fever has experienced a 30-fold increase in incidence worldwide over the past 50 years and shows no signs of slowing down [11]. Since the 1970s there have been several major outbreaks of dengue fever in southern China, including in Hainan, Guangxi, Fujian, Zhejiang, Yunnan and Guangdong provinces [12–14]. Due to climate change, the transmission of the dengue virus has spread gradually from southern tropical or subtropical regions to the surrounding northern and western regions, and even to the central China Henan Province with a generally warm temperate continental climate [15]. The most recent outbreak of dengue fever occurred in 2014 in Guangdong Province, with a total of 45,224 dengue fever cases and 6 deaths [12, 16, 17]. *Aedes albopictus* mosquitoes are regarded as the sole vector for dengue transmission in nearly all these epidemics [14, 18].

In China, *Ae. albopictus* is an indigenous species, closely associated with human migration, transportation, commerce and urbanization. It is the most important dengue vector species and has different susceptibilities to dengue virus in different geographical areas [19, 20]. Due to the lack of effective treatments or vaccines for dengue fever, vector control through chemical or biological measures targeting mosquitoes or their breeding sites is essential for dengue prevention. With the progressive spread of insecticide resistance, the threat of *Ae. albopictus* is growing, and development of efficient surveillance methods is more urgent than ever before [21, 22]. Population genetic studies of arthropod disease vectors can provide information about the transmission dynamics of specific pathogens, which aids in the design of strategies for controlling vector-borne disease epidemics [23, 24]. The recent waves of dengue outbreak in China highlight the need to improve our knowledge of *Ae. albopictus* population distribution and dynamics. Although scientists have studied the diversity of the *cox*1 gene in *Ae. albopictus* in several localities in China [9, 25–27], there have been no systematic studies of the genetic diversity of *Ae. albopictus* field populations and its cryptic species.

Cryptic species are defined as sibling species of two or more morphologically indistinguishable biological groups that are closely related and live in the same habitat [28]. Cryptic species may be medically important in vector-borne disease transmission, vector ecology and evolutionary biology. A number of new cryptic species have been identified in mosquito genera (Diptera: Culicidae), including *Culex* [29–31], *Anopheles* [32–39] and *Aedes* [40, 41]. In *Ae. albopictus*, a novel cryptic species has been reported in Vietnam [40], and the divergence between the cryptic species and *Ae. albopictus* was confirmed by analysis of nuclear ribosomal genes and mitochondrial genes. However, there are no reports of cryptic species of *Ae. albopictus* in other Asian countries, including China.

Natural infections of *Wolbachia* microbiota are common in *Ae. albopictus*, and the two *Wolbachia* biotypes, *w*AlbA and *w*AlbB, co-occur at a rate near 100% in many areas [42–46]. Maternally inherited *Wolbachia* bacteria can cause cytoplasmic incompatibility (CI) in many insect species, including *Ae. albopictus* mosquitoes [47, 48]. *Wolbachia* mediates antiviral protection of *Aedes* mosquitoes against a broad range of RNA viruses, including dengue, yellow fever, chikungunya and Rift Valley fever virus [49]. Understanding the distribution and prevalence of *Wolbachia* in *Ae. albopictus* and its cryptic species will provide useful information for vector control and host-symbiont coevolution.

In this study, we investigated the genetic diversity and population structure of *Ae. albopictus* from different climate regions in China, uncovered and molecularly identified the cryptic *Aedes* species and its polymorphism, and detected *Wolbachia* infection in the natural *Aedes* populations. The prevalence of *Wolbachia* endosymbiont was evaluated by multiplex PCR genotyping and DNA sequencing of individuals in the natural *Aedes* populations.

## Methods

### Sample collection

**Fig. 1:**
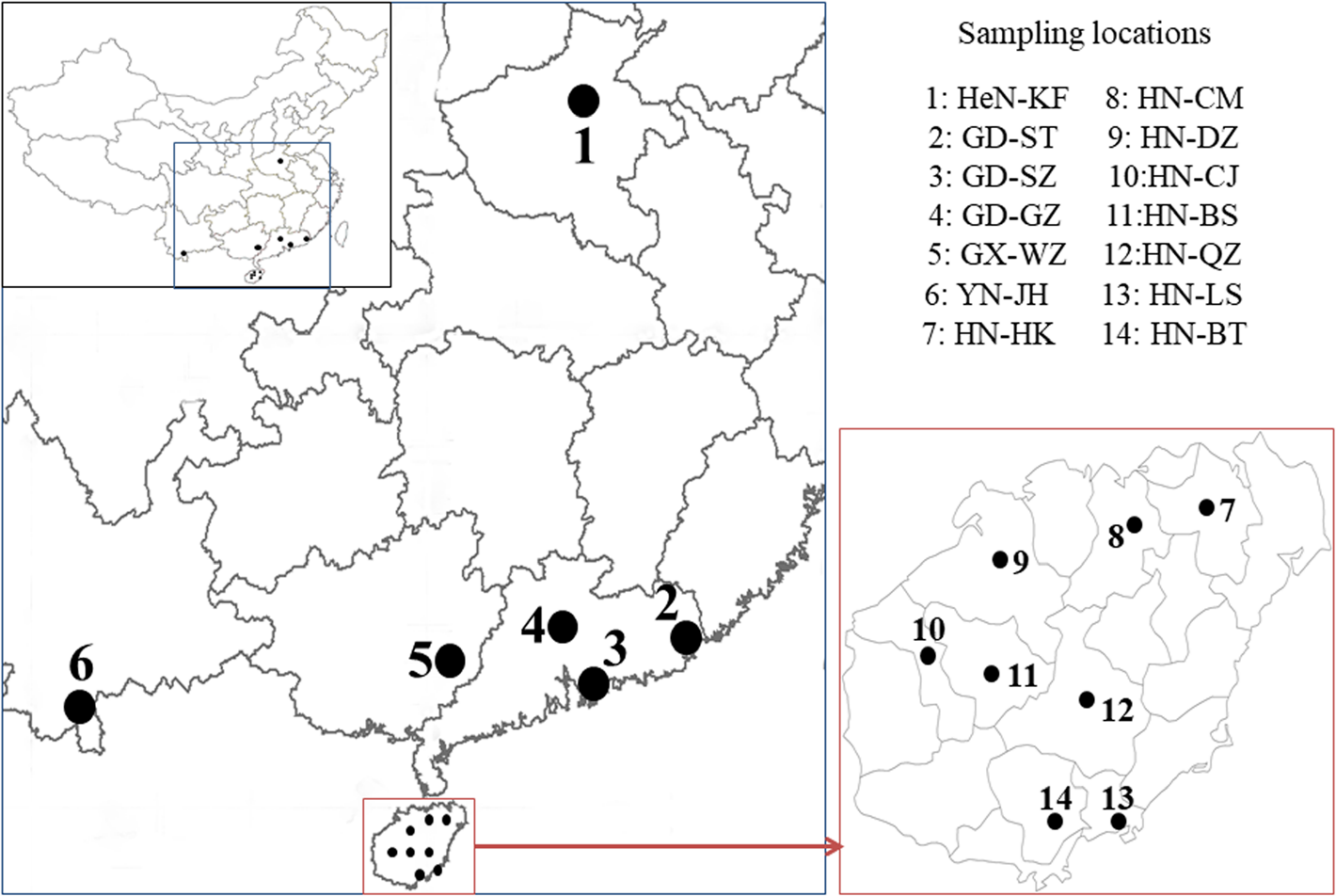
Locations of the 14 sampling sites. Site 1: Kaifeng (HeN-KF, 34°47'53" N, 114°18'05"E) in Henan Province. Sites 2–4: Shantou (GD-ST, 23°21'22"N, 116°40'40"E), Shenzhen (GD-SZ, 22°32'11"N, 113°55'32"E), and Guangzhou (GD-GZ, 23°07'54"N, 113°15'33"E) in Guangdong Province. Site 5: Wuzhou (GX-WZ, 23°53'43"N, 110°32'54"E) in Guangxi Province. Site 6: Jinghong (YN-JH, 22°00'10"N, 100°46'14"E) in Yunnan Province. Sites 7–14: Haikou (HN-HK, 20°02'47"N, 110°11'44"E), Chengmai (HN-CM, 19°44'25"N, 110°00'02"E), Danzhou (HN-DZ, 19°31'23"N, 109°34'36"E), Changjiang (HN-CJ, 19°17'60"N, 109°03'05"E), Baisha (HN-BS, 19°13'37"N, 109°26'51"E), Qiongzhong (HN-QZ, 19°02'06"N, 109°50'03"E), Lingshui (HN-LS, 18°30'27"N, 110°01'59"E) and Baoting (HN-BT, 18°38'27"N, 109°41'54"E) in Hainan Province. The map was created using the R package ‘*maptools’*, version: 0.9–2, URL: http://r-forge.r-project.org/projects/maptools/

Adult *Aedes* mosquito specimens were collected from April 2015 to October 2016 using BG-sentinel traps (Bioquip Products, Inc. California, USA) or electric aspirator mosquito catches [50] at 14 collection sites in five provinces in China: Henan, Guangdong, Guangxi, Yunnan and Hainan (Fig. 1). These sites are highly diverse in environmental conditions and most of them have experienced dengue epidemics in the past. The sampling site in Henan Province, located in central China, has a temperate climate with a distinct seasonality characterized by hot, humid summers and generally cold, windy and dry winters. The sampling sites in Guangdong and Guangxi provinces have a subtropical monsoon climate with long summers and year-round abundant precipitation. The sampling sites in Hainan and Yunnan provinces are tropical areas with a wet climate. Hainan Province had dengue epidemics in the late 1970s and early 1980s, however, no dengue epidemic has been reported from there since 1990. Guangdong Province has experienced multiple major dengue and chikungunya epidemics since 1980 and dengue has remained every year since 1994. In 2013, Guangdong, Yunnan and Henan provinces had dengue outbreaks. Dengue transmission occurred in southern China from July to November and the peak season is usually September and October whereas dengue epidemic in Henan Province is limited in summer from July to September, and showed earlier peaks and shorter epidemic periods [15]. *Aedes albopictus* mosquitoes are the primary vector of dengue virus across China, especially in urban areas. *Aedes aegypti* mosquitoes are only found in a small portion of southern China, including Hainan Province and small portions of Yunnan Province and southern tip of Guangdong Province [51]. Ten mosquito specimens from each collection site were used in the study. All mosquito specimens were morphologically identified under a stereomicroscope (Nikon) using morphological keys as described by Lu et al. [52]. All mosquito samples were stored at -20 °C prior to DNA extraction.

### PCR amplification and sequencing of mitochondrial DNA (mtDNA)

Total DNA was extracted from individual adult mosquitoes using the Insect DNA Kit (OMEGA Bio-Tek, D0926-01, Guangzhou, China) according to the manufacturer’s standard protocol. Extracted DNA was preserved at -20 °C until molecular analysis. The mitochondrial gene cytochrome *c* oxidase subunit 1 (*cox*1) was used to examine sequence polymorphism among mosquito samples. PCR was performed to amplify a 651 bp fragment of the 5' *cox*1 region of mtDNA using the DNA primer pairs LCOI490 (5'-GGT CAA CAA ATC ATA AAG ATA TTG G-3') and HCO2198 (5'-TAA ACT TCA GGG TGA CCA AAA AAT CA-3') [53, 54]. PCR amplification was performed in a 25 μl reaction volume with 12.5 μl GoTaq Green Master Mix (Promega, Guangzhou, China), 1 μl each of the forward and reverse primers at 10 μmol/l, 2 μl of template DNA and sufficient nuclease-free water to make 25 μl. PCR conditions were as follows: an initial denaturation at 94 °C for 1 min followed by five cycles of 94 °C for 40 s (denaturation), 45 °C for 40 s (annealing), and 72 °C for 1 min (extension); 30 cycles of 94 °C for 40 s (denaturation), 53 °C for 40 s (annealing), and 72 °C for 1 min (extension); and a final extension at 72 °C for 5 min. The amplified fragments were run on a 1% agarose gel to check integrity, stained with ethidium bromide and analyzed under UV light. PCR products were purified using a gel extraction kit (OMEGA Bio-Tek, D2500-02) and sequenced with PCR primers in both directions using the ABI 3730XL automatic sequencer (Applied Biosystems, Guangzhou, China). The sequences of *cox*1 unique haplotypes were deposited to the GenBank database under the accession numbers KY765450-KY765506.

### PCR amplification and sequencing of ribosomal DNA (rDNA)

The internal transcribed spacer 2 (ITS2) region of ribosomal DNA was amplified from the DNA samples using the universal primers ITS2A (5'-ATC ACT CGG CTC GTG GAT CG-3') and ITS2B (5'-ATG CTT AAA TTT AGG GGG TAG TC-3'), which anneal to highly conserved sequences in the *5.8S* and *28S* rDNA genes flanking the entire ITS2 region [21, 55]. PCR amplification was performed in a 25 μl reaction volume with 12.5 μl GoTaq Green Master Mix (Promega, Guangzhou, China), 1 μl each of the forward and reverse primers at 10 μmol/l, 2 μl of template DNA (1~2 ng/μl), and sufficient nuclease-free water to make 25 μl. PCR conditions were as follows: an initial denaturation at 94 °C for 3 min followed by 30 cycles of 94 °C for 30 s, 55 °C for 30 s, and 72 °C for 1 min; and a final extension at 72 °C for 5 min. Purification and sequencing of the PCR products were the same as described above for the *cox*1 gene. The sequences of ITS2 unique haplotypes were submitted to the GenBank database under the accession numbers MF623839-MF623851.

### PCR detection of *Wolbachia* infection in mosquitoes

The *Wolbachia* infection status of individual mosquitoes was determined by PCR amplification of *Wolbachia* ribosomal DNA using primers specific for *Wolbachia 16S* rDNA (WF: 5'-CAT ACC TAT TCG AAG GGA TAG-3' and WR: 5'-AGC TTC GAG TGA AAC CAA TTC-3') [56]. To further classify infected mosquitoes by *Wolbachia* group, we amplified the *Wolbachia* surface protein gene (*wsp*) using *w*AlbA primers (328F: 5'-CCA GCA GAT ACT ATT GCG-3' and 691R: 5'-AAA AAT TAA ACG CTA CTC CA-3') for A group and *w*AlbB primers (183F: 5'-AAG GAA CCG AAG TTC ATG-3' and 691R: 5'-AAA AAT TAA ACG CTA CTC CA-3') for B group [57]. PCR amplification was performed in a 25 μl reaction volume with 12.5 μl GoTaq Green Master Mix (Promega, Guangzhou, China), 1 μl each of the forward and reverse primers at 10 μmol/l, 2 μl of template DNA, and sufficient nuclease-free water to make 25 μl. PCR conditions were as follows: an initial denaturation at 94 °C for 3 min followed by 30 cycles of 94 °C for 30 s, 55 °C for 30 s, and 72 °C for 1 min; and a final extension at 72 °C for 5 min. PCR-amplified fragments of 408 bp, 364 bp, and 509 bp for *16S* rDNA, *w*AlbA and *w*AlbB, respectively, were revealed under UV light after electrophoresis on 1% agarose gel. Negative and positive controls for the PCR assay were included in each run. To obtain the positive control, we sequenced PCR fragments from the *16S* rDNA and *wsp* genes and confirmed that the amplified PCR product was *Wolbachia* by using BLAST search to compare it with existing sequences in the NCBI database.

### Data analysis

The *cox*1 gene sequences from 140 mosquitoes were aligned using Clustal W multiple alignment in BioEdit (version 7.2.6.1) [58]. The number of segregating sites, haplotype diversity (*H*_d_), and nucleotide diversity (π) within each population were determined using DnaSP version 5 [59]. Pairwise sequence divergences were calculated using a Kimura 2-parameter (K2P) distance model in MEGA 7.0.20 [60]. The K2P model was used to make our results comparable with most other studies on mosquito DNA barcoding. To examine population expansion, we also performed neutrality tests for each population. Deviations from selective neutrality were tested using Fu’s *F*_s_ statistic [61] and Tajima’s *D* [62]. To determine the genealogical relationships among haplotypes, we constructed a haplotype network using a statistical parsimony algorithm implemented in TCS version 1.21 [63]. The minimum number of mutational steps between sequences was calculated with > 95% confidence. A haplotype network shows the haplotype frequencies in each population and their relatedness, which is useful in inferring the plausible geographical origin of a population [64]. Genetic differentiation among populations was estimated using Arlequin 3.5 [65]. Analysis of molecular variance (AMOVA) was conducted to determine the distribution of genetic variation within and among populations and among groups (tropical, subtropical, and temperate zone).

To examine the evolutionary relationships between the individuals of *Ae. albopictus* and its cryptic species, we performed phylogenetic analysis using sequences of the mtDNA *cox*1 gene and the rDNA ITS2 region. Sequences from different species of the family Culicidae were selected from GenBank and used to build the phylogenetic trees. The *cox*1 phylogenetic tree was built using sequences of *Ae. aegypti* (AF390098, AY056597 and AF425846), *Cx. tritaeniorhynchus* (KT851544), *Cx. pipiens* (KT851543), *Cx. tarsalis* (AF425847), *Ae. flavopictus* (KT358463 and LC054359) and *Ae. albopictus* and its cryptic species (KF406577, JQ728019, KY378914, KF406649, KX495909, KX495922, KX495910, JQ728198, KY378918 and KY378935). The ITS2 phylogenetic tree was built using sequences of *Ae. aegypti* (GU980956, M95126 and KF471584), *Ae. flavopictus* (AF353532 and AF353548), *Cx. pipiens* (U22131 and U33044), *Cx. quinquefasciatus* (GU562872) and *Ae. albopictus* and its cryptic species (KU497617, DQ168420, KX495928, AF305554, KY382426, KX495936, KX495942 and KX495949). The predominant haplotype sequences of *Ae. albopictus* (*cox*1: KY765450, KY765468 and KY765479; ITS2: MF623839, MF623840 and MF623841) and all the haplotypes of cryptic species identified in the study, together with above GenBank sequences, were aligned using MAFFT v7.31 [66]. Phylogenetic trees were constructed based on the aligned nucleotide sequences using the neighbor-joining method *via* the maximum composite likelihood substitution model in MEGA 7.0.20 [60]. The statistical significance of tree branching was tested by performing 1000 bootstrap replications [67].

## Results

### Genetic polymorphism of *Ae. albopictus* and its cryptic species

PCR amplification and sequencing of the mitochondrial *cox*1 gene resulted in a 651 bp fragment for each individual study subject, with no insertions or deletions. We compared the *cox*1 sequences with the existing sequences in the NCBI database by BLAST search. Of the 140 individuals, 129 sequences (92.1%) were identical or possessed > 98% similarity with *Ae. albopictus* (GenBank: KR068634) (Additional file 1: Table S1). The remaining 11 individuals (7.9%) were approximately 10% K2P divergent from *Ae. albopictus*, indicating the existence of cryptic species (namely, *Aedes* sp.) of *Ae. albopictus* in China (Additional file 2: Table S2). Of these 11 individuals, 9 were from samples collected in Wuzhou, Guangxi Province, with one each from Baisha and Baoting, Hainan Province. Since there is only one individual of *Ae. albopictus* in GX-WZ population and one each of *Aedes* sp. from HN-BS, and HN-BT populations, which is insufficient for population genetic analysis, these individuals were excluded from the analysis of the population genetic structure and genetic diversity. Thus, only 9 individuals from each of these three populations (GX-WZ, HN-BS and HN-BT) were included in the genetic polymorphism analysis (Table 1). A high level of genetic diversity (*S* = 33; *H*_d_ = 0.972; *π* = 1.327; *k* = 8.639) was found in the Wuzhou (GX-WZ) population (cryptic species) compared with the 13 *Ae. albopictus* populations. Varied levels of genetic diversity were identified among the *Ae. albopictus* populations (Table 1). The Haikou (HN-HK) population had the highest number of polymorphism sites (*S* = 18), the greatest haplotype diversity (*H*_d_ = 0.978), and the highest average number of nucleotide differences (*k* = 4.333), followed by the Kaifeng (HeN-KF) population (*k* = 3.356). The three populations from Guangdong Province (GD-SZ, GD-ST and GD-GZ) had relatively low genetic diversity compared with populations from the other provinces. Varied genetic diversity was also found in the 8 populations from Hainan Province, with nucleotide diversity (*π*) ranging from 0.123 in specimens from Qiongzhong to 0.666 in specimens from Haikou. Tajima’s *D* tests for all study populations were not statistically significant (Table 1), indicating that the populations are in genetic equilibrium, consistent with the neutral mutation hypothesis. Likewise, Fu’s *F*_s_ test was not statistically significant and rejected the population expansion/bottleneck model for all study localities, with the exception of one Yunnan population (YN-JH, *F*_s_ = -4.738, *P* < 0.01) and two Hainan populations (HN-HK, *F*_s_ = -4.086, *P* < 0.05; HN-BT, *F*_s_ = -4.034, *P* < 0.05) (Table 1).

**Table 1:** Genetic polymorphism and neutrality tests of *Aedes albopictus* and its cryptic species in China

Province	Locality	Name	Species	*n*	*S*	h	*H*_d_	π	*k*	Tajima’s *D*	Fu's *F*_s_
Henan	Kaifeng	HeN-KF	*Ae. albopictus*	10	15	5	0.756	0.515	3.356	-1.691	0.584
Guangdong	Shantou	GD-ST	*Ae. albopictus*	10	5	5	0.667	0.178	1.156	-1.388	-1.896
	Shenzhen	GD-SZ	*Ae. albopictus*	10	2	2	0.2	0.061	0.4	-1.401	0.586
	Guangzhou	GD-GZ	*Ae. albopictus*	10	3	4	0.644	0.116	0.756	-1.034	-1.466
Guangxi	Wuzhou	*GX-WZ*	Cryptic *Aedes* sp.	9	33	8	0.972	1.327	8.639	-1.559	-1.358
			*Ae. albopictus*	1	–	–	–	–	–	–	–
Yunnan	Jinghong	YN-JH	*Ae. albopictus*	10	8	8	0.933	0.335	2.178	-0.992	-4.738**
Hainan	Haikou	HN-HK	*Ae. albopictus*	10	18	9	0.978	0.666	4.333	-1.489	-4.086*
	Chengmai	HN-CM	*Ae. albopictus*	10	5	5	0.8	0.205	1.333	-0.985	-1.547
	Danzhou	HN-DZ	*Ae. albopictus*	10	5	5	0.867	0.294	1.911	0.326	-0.706
	Changjiang	HN-CJ	*Ae. albopictus*	10	10	7	0.867	0.447	2.911	-0.782	-2.134
	Baisha	HN-BS	*Ae. albopictus*	9	4	5	0.833	0.179	1.166	-0.843	-2.109
			Cryptic *Aedes* sp.	1	–	–	–	–	–	–	–
	Qiongzhong	HN-QZ	*Ae. albopictus*	10	4	3	0.378	0.123	0.8	-1.667	0.058
	Lingshui	HN-LS	*Ae. albopictus*	10	4	5	0.8	0.164	1.067	-0.943	-2.096
	Baoting	HN-BT	*Ae. albopictus*	9	12	8	0.972	0.503	3.277	-1.221	-4.034*
			Cryptic *Aedes* sp.	1	–	–	–	–	–	–	–

*Abbreviations*: *n*, number of samples; *S*, number of segregating sites; h, number of haplotypes; *H*_d_, haplotype diversity; π, nucleotide diversity (× 10^2^, average number of nucleotide differences per site); *k*, average number of nucleotide differences

**P < 0.01

*0.01 < *P* < 0.05

**Fig. 2:**
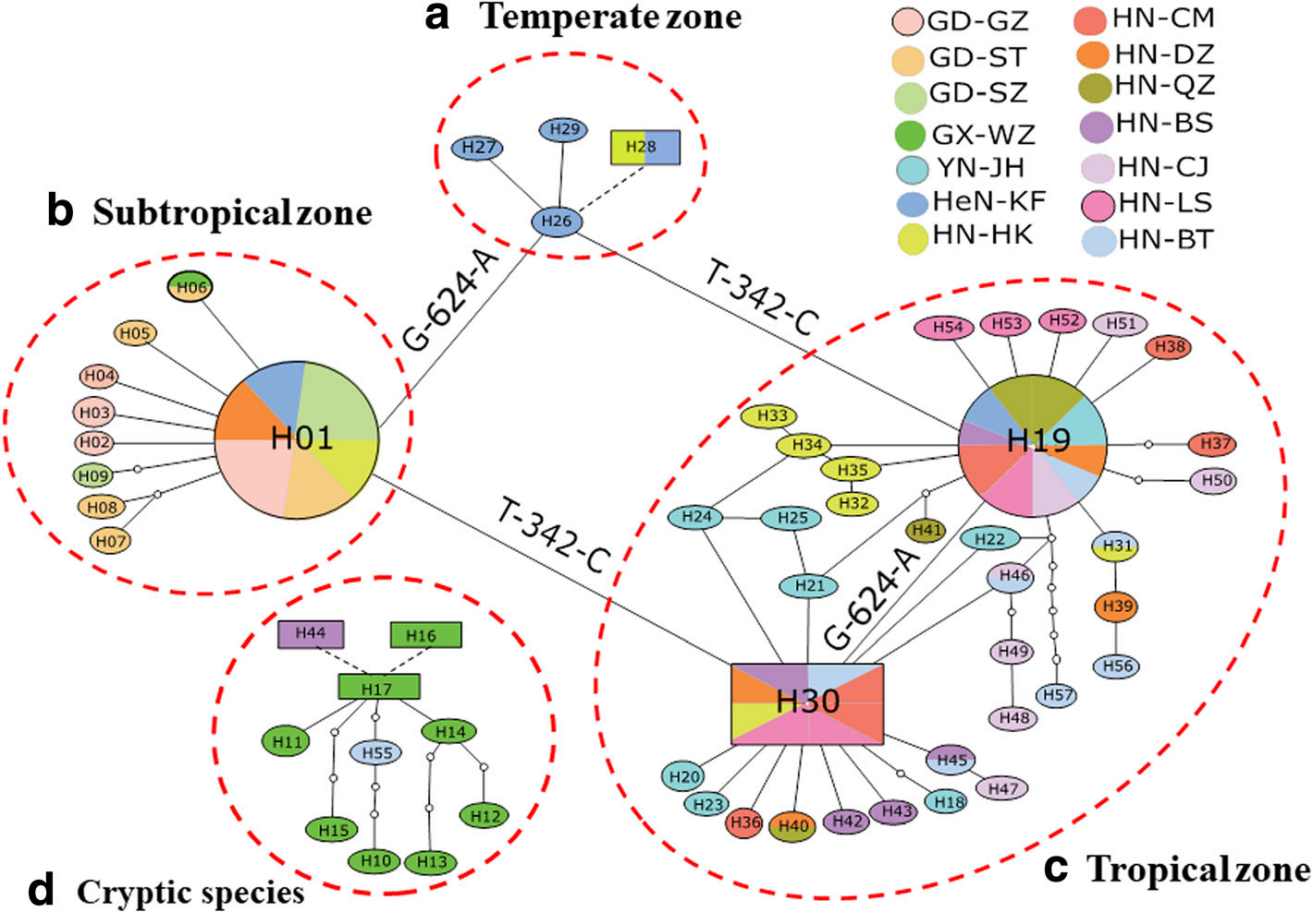
*Cox*1 haplotype networks showing the genealogical relationships. **a**-**c** *Aedes albopictus*. **d** Cryptic *Aedes* species. Each haplotype is represented by a pie chart with size proportional to its frequency in each population. A black dotted line indicates that a mutation step could not be determined between haplotypes at probability of parsimony above the 0.95 limit

A total of 57 haplotypes of mtDNA *cox*1 were detected in the 140 specimens, including 47 haplotypes derived from 129 *Ae. albopictus* mosquitoes and 10 haplotypes derived from cryptic species (Additional file 3: Table S3, GenBank: KY765450–KY765506). Three predominant haplotypes were identified in *Ae. albopictus* populations: H01 (21.5%) from Guangdong Province, H19 (22.1%) from Yunnan and Hainan provinces, and H30 (10%) from Hainan Province. Other haplotypes were either unique to a specific population or had a limited geographical distribution (Additional file 3: Table S3). To determine the relationships among the samples, we constructed a median-joining network using haplotypes based on sequence variation. Haplotypes were connected when the probability of parsimony was at least 0.95. Two networks were constructed based on all the 57 haplotypes, one based on haplotypes from *Ae. albopictus* (Fig. 2a-c) and the other based on haplotypes from the cryptic species (Fig. 2d). Three haplotypes could not be connected to the networks at a 95% confidence level: H28 for *Ae. albopictus*, and H16 and H44 for the cryptic species. The *Ae. albopictus* haplotypes can be classified into three clusters corresponding to three climate zones (tropical, subtropical and temperate) as shown in Fig. 2a-c. Haplotype H01 from Guangdong Province (subtropical) was connected to haplotypes from Hainan Province (tropical) through haplotype H30 by one mutation step at nucleotide position 342 (T-342-C), and to haplotypes from Henan Province (temperate) through H26 by one mutation step at nucleotide position 624 (G-624-A). Similar patterns were also observed for haplotype H19 from Yunnan and Hainan provinces (tropical zones). These results may imply a multiple origin for *Ae. albopictus* populations in China. In the cryptic species, haplotype H17 had at least 4 connections with other haplotypes, suggesting it as a potential ancestral haplotype (Fig. 2d).

**Fig. 3:**
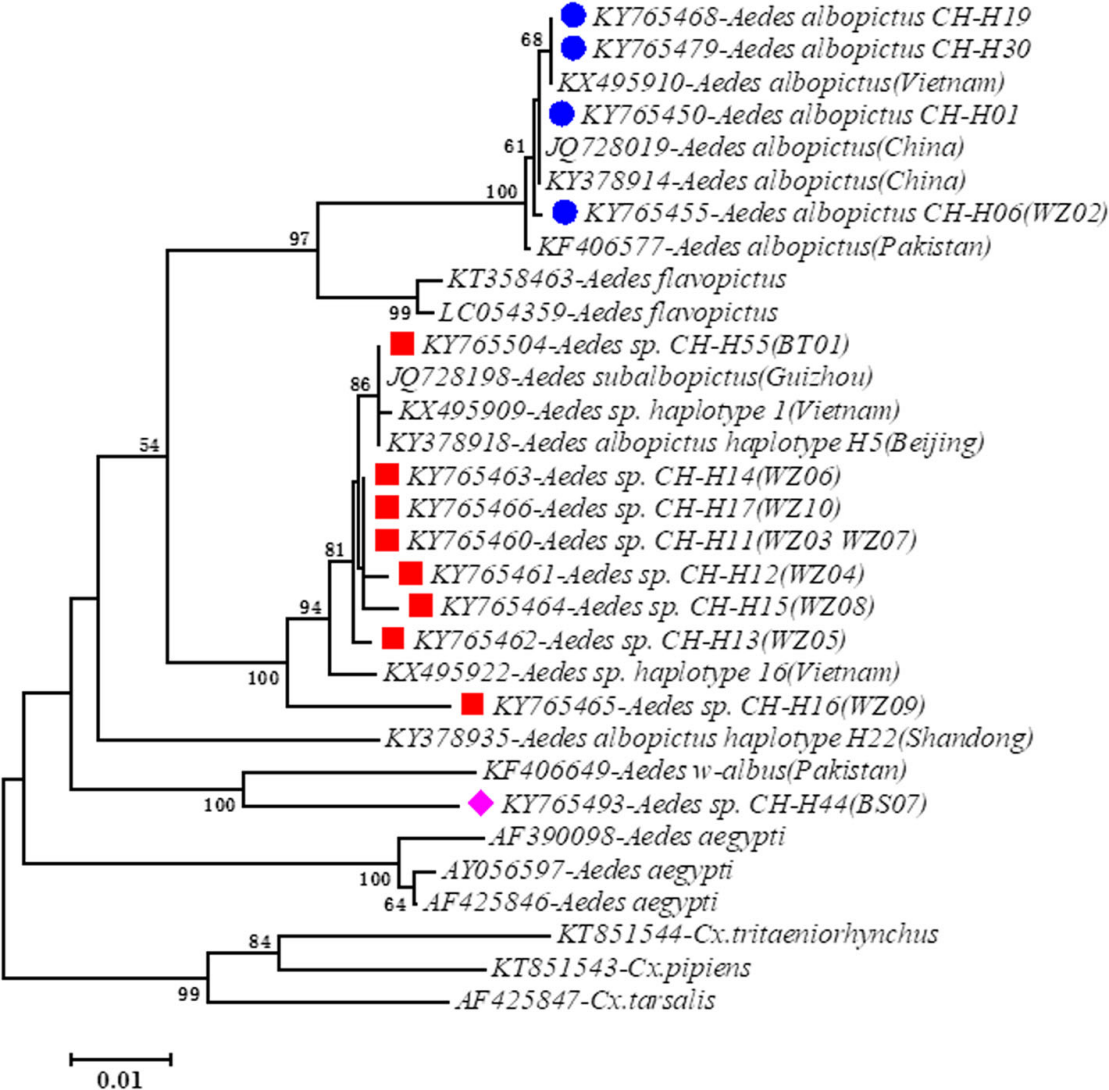
Phylogenetic analysis based on *cox*1 haplotype variation. Accession numbers of haplotypes marked with color symbols were identified in the current study; others were retrieved from GenBank. Haplotypes marked with a solid blue circle are associated with *Ae. albopictus*. Haplotypes marked with a red square or a purple diamond are associated with cryptic species of the *Ae. albopictus* subgroup. Neighbor-joining trees were constructed *via* the maximum composite likelihood substitution model using MEGA (version 7.0). Numbers at branches represent bootstrap values of 1000 replicates (values > 50 are shown). The scale-bar shows the number of nucleotide substitutions per site

**Fig. 4:**
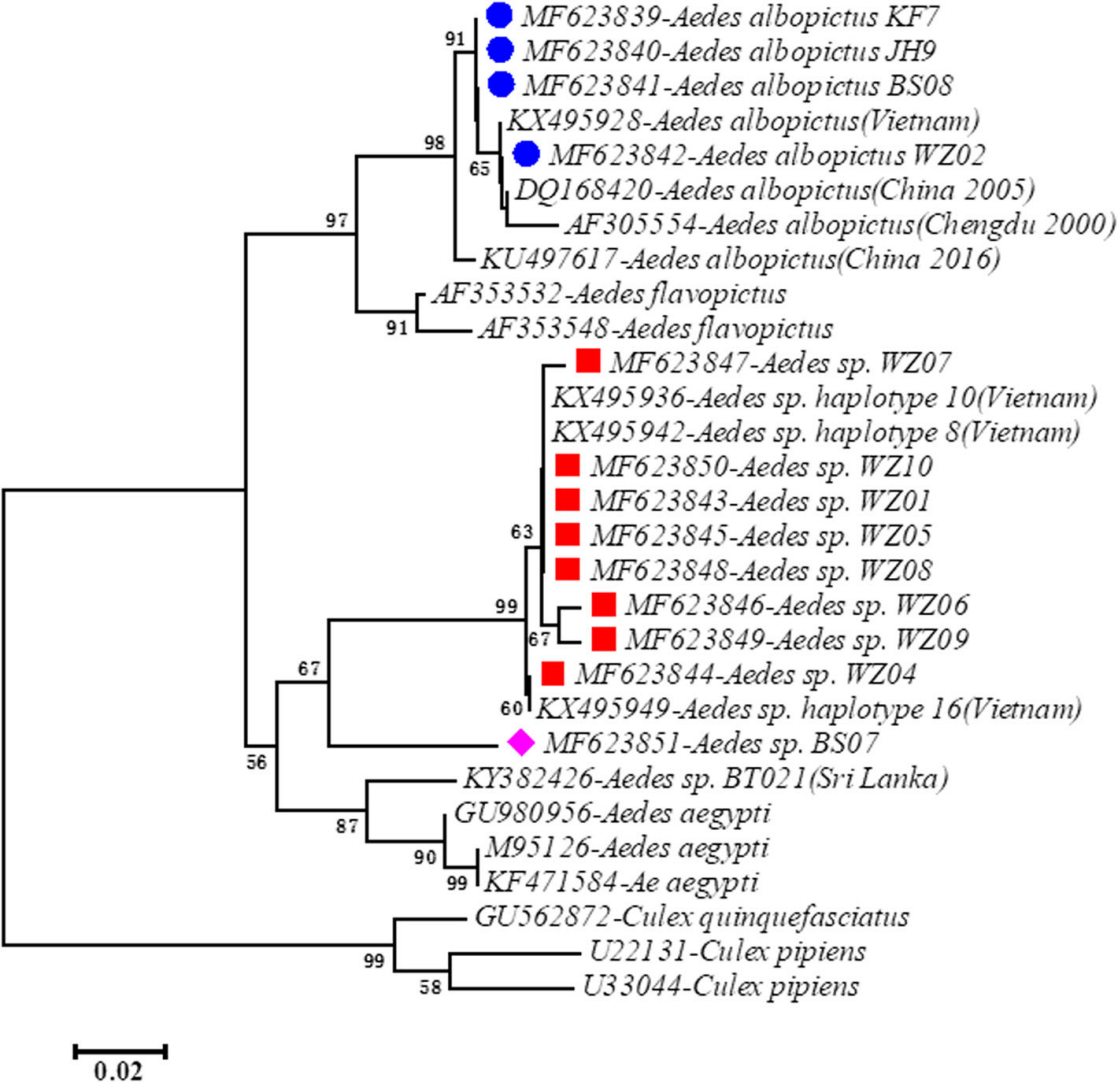
Phylogenetic analysis based on ITS2 haplotype variation. Accession numbers of haplotypes marked with color symbols were identified in the current study; others were retrieved from GenBank. Haplotypes marked with a blue circle are associated with *Ae. albopictus*. Haplotypes marked with a red square or a purple diamond are associated with cryptic species of the *Ae. albopictus* subgroup. Neighbor-joining trees were constructed *via* the maximum composite likelihood substitution model using MEGA (version 7.0). Numbers at branches represent bootstrap values of 1000 replicates (values > 50 are shown). The scale bar shows the number of nucleotide substitutions per site

A phylogenetic tree based on *cox*1 sequence variation indicated three clades, which were assigned to three different species (Fig. 3). The first clade (solid blue circles in Fig. 3) corresponds to *Ae. albopictus* from China and Vietnam, the second clade (red squares) corresponds to cryptic *Aedes* species previously identified in Vietnam, and the third clade (purple diamond) corresponds to cryptic *Aedes* species previously identified in Pakistan. The haplotype *Aedes* sp. CH-H44 (BS07) (purple diamond) is a different cryptic species compared to the haplotypes from Wuzhou, Guangxi Province (red squares). A similar pattern was observed in the phylogenetic tree based on ITS2 sequence variation (Fig. 4). The first clade (solid blue circles in Fig. 4) corresponds to *Ae. albopictus* from China and Vietnam and the second clade (red squares) corresponds to cryptic *Aedes* species previously identified in Vietnam. The ITS2 haplotype of *Aedes* sp. CH-H44 (BS07) (purple diamond) is also in different clade compared to the haplotypes from Wuzhou, Guangxi Province (red squares). The two taxa were distinguished using PCR length polymorphism at the ITS2 locus. *Aedes albopictus* had an amplicon size of ~580 bp, whereas the cryptic species had an amplicon size of ~415 bp, allowing for easy and accurate identification of the cryptic species through 1% agarose gel electrophoresis.

### Genetic differentiation of *Ae. albopictus* populations and cryptic population

Among the 13 *Ae. albopictus* populations, genetic differentiation was observed between populations from Henan Province (HeN-KF) and Guangdong Province (GD-ST, GD-SZ and GD-GZ) and populations from Yunnan and Hainan provinces. Thirty-four of 36 pairwise tests were significant at *P* < 0.05, and pairwise *F*_ST_ values ranged from 0.224 (between HeN-KF and HN-BT) to 0.750 (between GZ-SZ and HN-QZ), with an average of 0.411 (Table 2). No genetic differentiation was observed between populations within Hainan Province or between populations from Yunnan and Hainan provinces, indicating strong gene flow between these populations. All pairwise *F*_ST_ values of differentiation between cryptic population (GX-WZ) and the other 13 *Ae. albopictus* populations were highly significant and generally very high (*F*_ST_ values > 0.9) (Table 2). To further examine population structure and the extent of genetic variation between *Ae. albopictus* populations from tropical zones (Yunnan and Hainan provinces) and those from subtropical and temperate zones (Guangdong and Henan provinces), we conducted analysis of molecular variation (AMOVA) between the two groups of populations. Our results indicated a significant overall population structure in *Ae. albopictus* (*F*_ST_ = 0.38, *P* < 0.001). The majority of genetic variation (61.77%) was within populations, whereas approximately 34.21% was between the two groups, and only 4.01% of variation was among populations within groups (Table 3).

**Table 2:** Pairwise genetic differentiation (*F*_ST_) between *Ae. albopictus* populations and the cryptic population in China

	HeN-KF	GD-ST	GD-SZ	GD-GZ	YN-JH	HN-HK	HN-CM	HN-DZ	HN-CJ	HN-BS	HN-QZ	HN-LS	HN-BT
GD-ST	0.091												
GD-SZ	0.106	0.028											
GD-GZ	0.098	0.044	0.037										
YN-JH	0.260*	0.405*	0.463*	0.436*									
HN-HK	0.118	0.327*	0.360*	0.344*	0.059								
HN-CM	0.285*	0.521*	0.606*	0.557*	0.046	0.030							
HN-DZ	0.139	0.290*	0.358*	0.333*	0.054	0.042	0.057						
HN-CJ	0.236*	0.419*	0.466*	0.444*	0.071	0.026	0.008	0.080					
HN-BS	0.292*	0.488*	0.588*	0.539*	-0.007	0.082	0.012	0.035	0.044				
HN-QZ	0.347*	0.651*	0.750*	0.701*	0.182	0.028	0.048	0.183	0.081	0.227			
HN-LS	0.309*	0.573*	0.667*	0.620*	0.089	0.029	-0.034	0.092	0.035	0.070	0.007		
HN-BT	0.224*	0.348*	0.397*	0.377*	0.004	0.055	0.036	-0.003	0.010	-0.011	0.152	0.067	
GX-WZ^a^	0.905*	0.925*	0.931*	0.928*	0.917*	0.898*	0.923*	0.918*	0.911*	0.922*	0.927*	0.926*	0.905*

^a^*Aedes* sp. cryptic population

*Asterisks indicate significant values after Bonferroni correction (*P* < 0.05)

**Table 3:** Analysis of molecular variance (AMOVA) of two groups of populations of *Ae. albopictus* in China

Source of variation	df	SS	Variance components	Percentage of variation
Among groups	1	30.34	0.5234 Va	34.21
Among populations within groups	11	17.04	0.0614 Vb	4.01
Within population	115	108.68	0.9450 Vc	61.77
Total	127	156.06	1.5299	–

*Abbreviations*: *df* degrees of freedom, *SS* sum of squares

### PCR detection of *Wolbachia* infection in natural mosquito populations

**Fig. 5:**
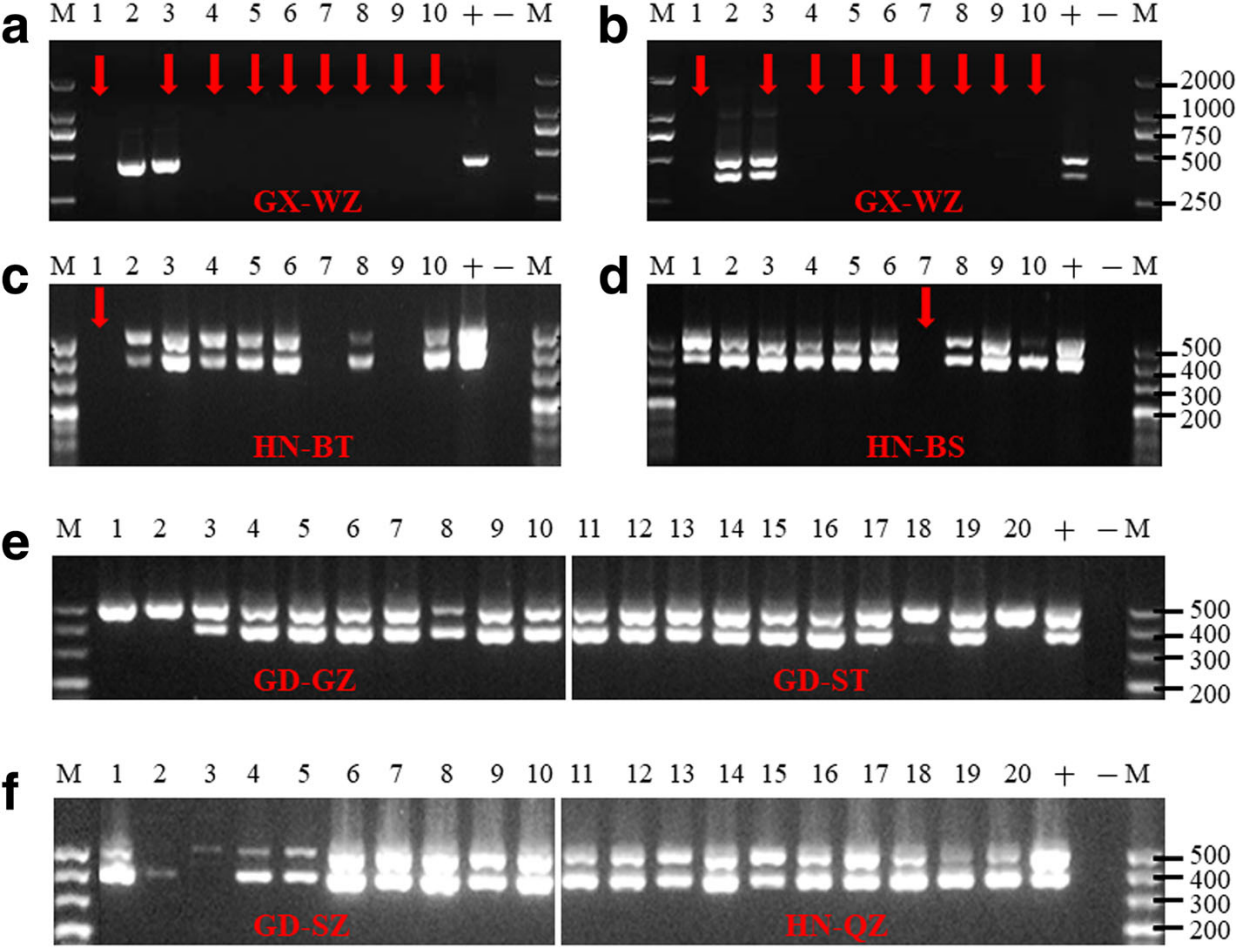
Example of banding patterns of agarose gel electrophoresis. PCR products amplified using primers for *Wolbachia*-specific *16S* rRNA gene (**a**) and *Wolbachia* surface protein gene (*wsp*) (**b**-**f**) in natural populations. Lanes 1–10: PCR products of 10 individuals from specific locations; Lanes “+” and “–”: positive and negative controls, respectively; Lane M: DNA ladder. Red arrows indicate cryptic *Aedes* species

*Wolbachia* infections were detected in all 14 of the *Ae. albopictus* populations. Infection rates ranged from 50% (HN-DZ) to 100% (in 8 populations) with an average of ~90% (Table 4), suggesting that *Wolbachia* is highly prevalent in *Ae. albopictus* in China. In the cryptic *Aedes* sp. species, however, *Wolbachia* infection was absent (HN-BT and HN-BS) or occurred at low frequency (11%, GX-WZ) (Fig. 5a-d). Most infected individuals were infected with both the *w*AlbA and *w*AlbB strains of *Wolbachia*; the average superinfection rate in the 14 *Ae. albopictus* populations was 70%, with a range from 10% (HN-DZ) to 90% (YN-JH, HN-CJ and HN-QZ). Single-strain *Wolbachia* infections were found in five populations for *w*AlbA and eight populations for *w*AlbB, with low prevalence (< 20%) (Fig. 5e, f) except in HN-CM (70% *w*AlbA) (Table 4).

**Table 4:** Results of PCR screening for *Wolbachia* infection in natural *Ae. albopictus* populations and cryptic *Aedes* species in China

Province	Locality	Name	Species	Total female	*16S* rRNA *n* (%)	*w*AlbA *n* (%)	*w*AlbB *n* (%)	Type A and type B *n* (%)	Uninfected *n* (%)
Henan	Kaifeng	HeN-KF	*Ae. albopictus*	10	9 (90.0)	0 (0)	2 (20.0)	7 (70.0)	1 (10.0)
Guangdong	Shantou	GD-ST	*Ae. albopictus*	10	10 (100)	0 (0)	2 (20.0)	8 (80.0)	0 (0)
	Shenzhen	GD-SZ	*Ae. albopictus*	10	10 (100)	1 (10.0)	1 (10.0)	8 (80.0)	0 (0)
	Guangzhou	GD-GZ	*Ae. albopictus*	10	10 (100)	0 (0)	2 (20.0)	8 (80.0)	0 (0)
Guangxi	Wuzhou	GX-WZ	Cryptic *Aedes* sp.	9	1 (11.1)	0 (0)	0 (0)	1 (11.1)	8 (88.9)
			*Ae. albopictus*	1	1 (100)	0 (0)	0 (0)	1 (100)	0 (0)
Yunnan	Jinghong	YN-JH	*Ae. albopictus*	10	10 (100)	0 (0)	1 (10.0)	9 (90.0)	0 (0)
Hainan	Haikou	HN-HK	*Ae. albopictus*	10	9 (90.0)	0 (0)	2 (20.0)	7 (70.0)	1 (10.0)
	Chengmai	HN-CM	*Ae. albopictus*	10	10 (100)	7 (70.0)	0 (0)	3 (30.0)	0 (0)
	Danzhou	HN-DZ	*Ae. albopictus*	10	5 (50.0)	2 (20.0)	2 (20.0)	1 (10.0)	5 (50.0)
	Changjiang	HN-CJ	*Ae. albopictus*	10	10 (100)	0 (0)	1 (10.0)	9 (90.0)	0 (0)
	Baisha	HN-BS	*Ae. albopictus*	9	9 (100)	1 (11.1)	0 (0)	8 (88.9)	0 (0)
			Cryptic *Aedes* sp.	1	0 (0)	0 (0)	0 (0)	0 (0)	0 (0)
	Qiongzhong	HN-QZ	*Ae. albopictus*	10	10 (100)	1 (10.0)	0 (0)	9 (90.0)	0 (0)
	Lingshui	HN-LS	*Ae. albopictus*	10	6 (60.0)	0 (0)	0 (0)	6 (60.0)	4 (40.0)
	Baoting	HN-BT	*Ae. albopictus*	9	7 (77.8)	0 (0)	0 (0)	7 (77.8)	2 (22.2)
			Cryptic *Aedes* sp.	1	0 (0)	0 (0)	0 (0)	0 (0)	0 (0)

## Discussion

*Aedes albopictus*, one of the cryptic species of the *Ae. albopictus* subgroup, is an important vector for public health. It is highly invasive and is the most widely distributed mosquito species in the world. *Aedes albopictus* originated at the edges of forests and bred in natural habitats, but it has adapted to being a domestic mosquito [13]. Today, the species can be found throughout tropical, subtropical and temperate zones in China. Cryptic species often occur in sympatry and are so similar they cannot be distinguished *via* traditional species identification using morphological keys [68, 69]. With the advent of molecular diagnosis technology and relatively inexpensive DNA sequencing technology, the discovery of cryptic species has become common in many insect groups [70]. However, little is known about the cryptic species within the *Ae. albopictus* subgroup in China. To the best of our knowledge, this is the first report describing the discovery of phylogenetically divergent cryptic species living in sympatry with *Ae. albopictus* in China. In this study, among 14 study populations collected across the tropical, subtropical and temperate climate zones of China, we found three populations in southern China in which cryptic species of *Ae. albopictus* coexisted in sympatry. Furthermore, multiple haplotypes of cryptic species were present in the Wuzhou population in Guangxi Province within the subtropical zone, and probably a novel cryptic species (KY765493) existed in the Baisha population in the tropical area of Hainan Province. Further investigation is needed to confirm the reproductive isolation of these species. These results may have important implications for vector control and for understanding the evolutionary processes of the species. For example, if the cryptic species is a novel disease vector with different biting or resting behaviors, current vector control interventions that target on species-specific vector behavior could lead directly to programme failure and thus these vector control strategies need to be adjusted.

The distribution of *Aedes* mosquito species is influenced by climatic, environmental and geographical factors, as well as by human behavior [71–73]. In this study, we found significantly higher genetic diversity in cryptic species populations than in *Ae. albopictus* populations. For example, the cryptic species population in Wuzhou (GX-WZ) has 33 segregation sites, with much higher nucleotide diversity and the highest average number of nucleotide differences compared with the *Ae. albopictus* populations. The high genetic diversity of cryptic species, as well as their coexistence with *Ae. albopictus*, may be explained by environmental heterogeneity in these densely forested, mountainous areas with a low level of gene flow and random genetic drift. The different population structure between tropical (Hainan and Yunnan) and subtropical (Guangdong)/temperate (Henan) populations of *Ae. albopictus* might be due to selective pressures exerted by specific climate, environment and human activities. The mountainous areas of Yunnan and central Hainan provinces have lower human population density and more complex tropical environments than the densely populated Guangdong (coastal) and Henan (plain) provinces. The association between genetic population structure and climate/environment has also been observed in other *Ae. albopictus* populations [19, 74, 75]. Gene flow between the two genetic clusters (tropical and subtropical/temperate) appeared to be restricted due to geographical isolation, which was also evidenced in the *cox*1 haplotype network analysis. Interestingly, both of the major tropical-zone haplotypes, H30 and H19, were just one mutation step (C-342-T) from subtropical (Guangdong Province) and temperate (Henan Province) haplotypes, and the same mutation step (G-624-A) was found between populations within tropical zones as well as between populations from subtropical and temperate zones. These results suggest that climate, geography, environment and human activity all play important roles in *Ae. albopictus* population structure.

The maternally transmitted endosymbiotic bacterium *Wolbachia* is known to have an important impact on host reproduction in many insects, including mosquitoes (Diptera: Culicidae) [47, 76, 77]. *Wolbachia* can inhibit human pathogens transmitted by mosquitoes, including dengue virus [78–80], yellow fever [81], filarial nematodes [82, 83], malaria parasites [84–86] and Zika virus [87]. Our results indicate significant variations in the frequency of *Wolbachia* infection in *Ae. albopictus* populations. Populations from Guangdong, Yunnan, and many sites in Hainan Province had a 100% infection rate, followed by a 90% infection rate in Henan Province in central China. Such high prevalence of *Wolbachia* has also been observed in other Asian countries, including Malaysia [43, 88], Thailand [89], India [90] and Sri Lanka [91]. In our study, most populations were naturally infected with two *Wolbachia* strains (*w*AlbA and *w*AlbB) at high frequencies, suggesting that superinfection is common in *Ae. albopictus*, as observed in other studies [44, 88, 89, 92]. Interestingly, a relatively low prevalence of *Wolbachia* was also observed in three *Ae. albopictus* populations (HN-DZ, HN-LS, and HN-BT) from Hainan Province, in which no more than 50% of the individuals were not detected with *Wolbachia* infection, indicating that there was variation in natural *Wolbachia* infection in these *Ae. albopictus* populations. Furthermore, no *Wolbachia* infection was detected in the *Aedes* cryptic species from Hainan Province, and *Wolbachia* infection was detected in just one individual of the cryptic species from Guangxi Province. A similar pattern was observed in the cryptic species in Vietnam [40], suggesting that the cryptic species may be resistant to *Wolbachia* infection or that *Wolbachia* are present at low cell density in the cryptic species that cannot be detected by PCR [93]. In addition, cryptic species may prevent *Wolbachia* introgression by reproductive isolation and maintaining ancestral levels of mitochondrial diversity. *Wolbachia* induced cytoplasmic incompatibility and mitochondrial selective sweep have been observed in the *Ae. albopictus* and other mosquito species [29, 48]. Further studies are needed to confirm the reproductive isolation between *Ae. albopictus* and its cryptic species.

## Conclusions

Our results indicated that the genetic diversity and population structure of *Ae. albopictus* between tropical, subtropical and temperate zones in China appeared to be separated by a single mutation step at the mitochondrial DNA barcoding *cox*1 gene. Sympatric, cryptic sibling species might be common in the *Ae. albopictus* subgroup in China. The prevalence of high-level *Wolbachia* infection in most of the *Ae. albopictus* populations, and the absence or low prevalence of *Wolbachia* in the sympatric cryptic species, possibly due to *Wolbachia*-induced genetic hitchhiking or selective sweep that has created a barrier to gene flow among the species. Elucidating the mechanisms of the observed absence or low prevalence of *Wolbachia* in sympatric cryptic species may provide insight toward the development of new vector control strategies. Finally, this study will have important implications for disease vector-based control programs, *Wolbachia*-based disease control strategies, and host evolutionary biology. Further study is needed to investigate the potential for arbovirus infection and disease transmission in the emerged cryptic species.

## Additional files

Additional file 1: **Table S1.** K2P divergence of *cox*1 sequences in *Ae. albopictus* from China. (XLSX 76 kb)

Additional file 2: **Table S2.** K2P divergence of *cox*1 sequences in *Aedes *sp. identified in China. (XLSX 9 kb)

Additional file 3: **Table S3.** *cox*1 haplotype distribution in the 14 mosquito populations from China. (XLSX 14 kb)

## Abbreviations

AMOVA: Analysis of molecular variance
*cox*1: cytochrome *c* oxidase subunit 1
GD-GZ: Guangzhou
GD-ST: Shantou
GD-SZ: Shenzhen
GX-WZ: Wuzhou
HeN-KF: Kaifeng
HN-BS: Baisha
HN-BT: Baoting
HN-CJ: Changjiang
HN-CM: Chengmai
HN-DZ: Danzhou
HN-HK: Haikou
HN-LS: Lingshui
HN-QZ: Qiongzhong
ITS2: DNA internal transcribed spacer region 2
K2P: Kimura 2-parameter
mtDNA: mitochondrial DNA
rDNA: ribosomal DNA
*wsp*: *Wolbachia* surface protein gene
YN-JH: Jinghong
